# Sensitivity, Specificity, Negative and Positive Predictive Values of Adenosine Deaminase in Patients of Tubercular and Non-Tubercular Serosal Effusion in India

**DOI:** 10.4021/jocmr2010.05.289w

**Published:** 2010-06-15

**Authors:** Bharat Kumar Gupta, Vinay Bharat, Debapriya Bandyopadhyay

**Affiliations:** a Department of Biochemistry, Subharti Medical College, S. V. S. University, Meerut, India; b Department of Pathology, Subharti Medical College, S. V. S. University, Meerut, India

## Abstract

**Background:**

In India, tuberculosis is an endemic disease. Delay in diagnosis results in poor prognosis and fast spread of the disease. The objective of the present study is to look for an effective and acceptable diagnostic test, which may be helpful to initiate early treatment to improve prognosis and reduce spread.

**Methods:**

Three hundred and thirty patients with pleural, ascitic, meningeal and synovial effusion were selected and divided depending upon the etiology and the involvement of serosal membranes. Serosal aspirated fluid was subjected to biochemical tests and adenosine deaminase estimation. Cutoff taken is above 40 for pleural, peritoneal or synovial fluid and above 10 for CSF.

**Results:**

In cases of pulmonary and extra-pulmonary disease, sensitivity was 92.80% and 94.29%; specificity 90.00% and 92.16%; positive predictive value 92.86% and 89.00%; and negative predictive value 90.00% and 95.92% respectively.

**Conclusions:**

Adenosine deaminase estimation is not only a fairly sensitive and specific test (more than 90%), helpful in differentiating tubercular from non-tubercular etiology both in pulmonary and extra-pulmonary disease, but is also simple, inexpensive and rapid. For this reason this test may help in early diagnosis, improve the prognosis and reduce spread of disease and sequlae.

**Keywords:**

Adenosine deaminase; Serosal effusion; Tubercular; Non-tubercular; Pulmonary; Extra-pulmonary

## Introduction

Tuberculosis is one of the commonest chronic infectious diseases, which is highly endemic in India and five lakh patients die every year [1]. It usually affects lungs but cases of extra-pulmonary tuberculosis are not rare. Delay in diagnosis and in initiating treatment results in poor prognosis and sequlae in up to 25% of cases [2]. In a large percentage of cases, involvement of pleura, peritoneum, meninges and synovial membranes is also common. Pulmonary Tuberculosis (TB) can be confirmed by sputum examination and diagnosed easily, but diagnosing extra-Pulmonary TB becomes frequently difficult since the specificity and sensitivity of non-invasive methods is very low.

When aspirated serosal fluids were analyzed for direct evidence of disease, acid fast bacilli (AFB) positivity rate is very low and this process is time consuming also. AFB culture is expensive and requires long incubation period and is positive in less than 25% cases (Rafae Laniado-Laborin). Direct examination of pleural fluid by Zeihl-Neelsen staining requires bacillary densities of 10,000/mL [3] and, therefore, detects AFB in less than 10% of cases. Elisa, PCR, Tub IgG and IgM, and interferon are very expensive tests and beyond the reach of the general population in poor countries.

Adenosine deaminase (ADA) is an enzyme in the purine salvage pathway that catalyzes the conversion of adenosine and deoxyadenosine to inosine and deoxyinosine with the release of ammonia. It plays important role in differentiating lymphoid cells and is present in abundance in active T-lymphocytes whose concentration is inversely proportional to the degree of differentiation [4]. Its levels are ten times higher in T-lymphocytes than in erythrocytes. The enzyme activity increases during mitogenic and antigenic responses of lymphocytes, and T-lymphocyte blastogenesis can be inhibited by inhibitors of ADA. Likewise, a deficiency of adenosine deaminase is associated with severe defects in the cell-mediated and humoral arms of the immune system, predisposing the patient to opportunistic infections.

Tuberculous effusion is the result of a cell-mediated immune response to the presence of Mycobacterium tuberculii and is characterized by the accumulation of activated T-lymphocytes and macrophages. Elevated levels of ADA in tuberculous effusions have been noted by several authors. ADA is now being recognized as a marker of cell-mediated immunity particularly as a marker of T-lymphocyte activation.

Since ADA is increased in TB effusions and is an easy little-invasive investigation, it is frequently considered as a diagnostic aid in such cases with a sensitivity of 90 - 100% and specificity 89-100%. ADA levels have also been considered by several researchers to differentiate tubercular disease from non-tubercular [5-7]. The aim of the present study is to: (1) propose a simple, rapid and cost effective test for diagnosing tuberculosis and differentiating it from other etiologies both in pulmonary and extra-pulmonary disease with fairly good accuracy; (2) initiate early anti-tubercular treatment to improve prognosis and reduce spread and sequlae.

## Methods

Patients attending out and in-patient departments of chest and respiratory diseases, medicine, pediatrics and orthopedic surgery of Subharti Medical College and its rural and urban health Centers at Sarawani, Mahalwala, Khajoori and Multan Nagar at distant places in district Meerut of India from January 2007 to October 2009 with pleural, peritoneal, meningeal and synovial tubercular disease with serosal effusion were taken.

The project was approved by the ethical committee of our institution. After taking informed consent, 330 such patients were included in the present study. Detailed clinical history, physical examination, investigations, for example, Hb, T/DLC, ESR, GBP, CBC, SGOT, SGPT, PPD test and X-ray/ultrasound were done in all the cases. AFB in the sputum/fluid, AFB culture, ECG, cytology and biopsy with its histopathological examination were done in selective cases.

Effusions were aspirated and fluid was subjected to the biochemical examination and ADA activity testing. Any other relevant/necessary/required investigations were also carried out in specific case.

Presence of first or more than one of the following criteria was adopted to label a case as tuberculous: (1) Bacteriological confirmation of presence of Mycobacterium tuberculosis (direct smear or culture or histological finding); (2) Histopathology finding of caseous granulomas; (3) Radiological findings consistent with TB; (4) Clinical presentation consistent with TB with exclusion of other clinical considerations; (5) Definite clinical and radiological improvement in two months on administration of exclusive anti-tuberculosis treatment; (6) History of contact with current disease and positive reaction (more than 20 mm induration) to the 5 tuberculin unit (TU) purified protein derivative (PPD).

Following precautions were taken while performing the ADA and other routine tests on the fluids: (1) Fluids were collected in sterile containers; (2) Detailed gross examination which included volume, color, turbidity, any flakes etc. was noted; (3) Microscopic examination of the fluid was done for total and differential leukocyte count and also for any other abnormal cell; (4) Fluid was centrifuged, slide prepared from the residue, stained by Z - N staining and examined for AFB microscopically; (5) ADA test was done by Guisti and Galanti method [8] on the same day, the fluid was received. If a delay in performing the test was anticipated, the fluid was kept in the refrigerator at 2 - 4 ^o^C after adding glycerol to it, to avoid decrease in ADA level with the lapse of time at ambient temperature.

In case of readings beyond the linearity of the assay system, the test was repeated with appropriate dilutions of the samples and the result was calculated considering the dilution factor. In case of turbid effusions the fluids were centrifuged and the test was performed from the clear supernatant. And if the fluid was still found to be turbid, it was excluded from the study.

Reference values, as follows, were taken as per the recommendations of Microxpress, a division of Tulip Diagnostics (P) Ltd. India, of which chemicals and reagents were used. ADA levels above 40 U/L were taken as positive in samples from pleural, ascitic or synovial fluid while ADA levels above 10 U/L were taken as positive in CSF (Table 1).

**Table 1:** Reference Values of ADA in Various Body Fluids (U/L)

Type of Fluid	Negative	Positive
Serum/plasma/Pleural/Ascitic/synovial Fluids	40 or < 40	> 40
CSF	10 or < 10	> 10

## Results

Out of 330 aspirated samples, 72 were pulmonary (Pleural) and 258 were extra-pulmonary serosal effusions. Amongst extra-pulmonary, 108 were peritoneal (Ascitic), 120 were cerebrospinal and 30 were synovial fluids. These samples were divided according to the criteria set for tuberculosis: 42 samples were tubercular while 30 were non-tuberculous amongst pulmonary disease and amongst extra-pulmonary; 36 samples were tubercular while 72 were non-tubercular in abdominal disease; 54 samples were tubercular while 66 were non-tubercular in cerebrospinal disease; and 21 samples were tubercular while 9 were non-tubercular in musculoskeletal disease (Table 2).

**Table 2:** Table Showing Distribution of Cases According to the Initial Set Criteria

Type of Disease	Aspirated Fluid	Etiology; According to Set Criteria	Number of Cases
1. Pulmonary	Pleural (n = 72)	Tuberculous	42
		Non tuberculous	30
2. Extra-Pulmonary	Ascitic / Peritoneal (n = 108)	Tuberculous	36
		Non tuberculous	72
	CSF (n = 120)	Tuberculous	54
		Non tuberculous	66
	Synovial (n = 30)	Tuberculous	21
		Non tuberculous	09
Total			330

Only 14/42 (33.34%) amongst pulmonary tubercular disease and 9/36 (25%) amongst peritoneal tubercular disease were confirmed by direct evidence of AFB while none in synovial fluid and CSF showed the evidence of AFB positivity. In the present study, only 23/153 (15.03%) patients could be conformed by evidence of AFB (Table 3).

**Table 3:** Table Showing ADA and AFB Positivity in Tubercular Group

Type of Tubercular Disease	Aspirated Fluid	Etiology; No. of Tubercular Cases According to Set Criteria	AFB Positive Cases
Pulmonary (n = 42)	Pleural (n = 72)	42	14
Extra-Pulmonary (n = 111)	Ascitic/Peritoneal (n = 108)	36	09
	CSF (n = 120)	54	nil
	Synovial (n = 30)	21	nil
Total		153	23

ADA levels in tubercular pleural fluid ranged between 40.4 and 136 with a median value of 88 while in non-tubercular pleural effusions it ranged between 4.8 and 38 with a median value of 21.5. ADA levels in tubercular ascitis ranged between 48 and 180 with a median level of 110, and in non- tubercular ascitis it ranged between 3.3 and 40 with a median value of 16. ADA levels in tubercular joint effusions ranged between 46 and 156 with a median level of 88, and in non- tubercular joint effusions it ranged between 25 and 32.6 with a median value of 27.2. ADA levels in tubercular CSF ranged between 10.2 and 110 with median value of 24, and in non-tubercular CSF value was found to be between 2 and 10 with a median value of 6.4 (Table 4).

**Table 4:** Table Showing Range of ADA With Median Corresponding to Etiology

Type of Fluid	Cut Off	Range U/L	Median
Pleural	> 40	40.4 - 136	88
	≤ 40	4.8 - 38	21.5
Ascitic/Peritoneal	> 40	48 - 180	110
	≤ 40	3.3 - 40	16
Synovial	> 40	46 - 156	88
	≤ 40	25 - 32.6	27.2
CSF	> 10	10.2 - 110	24
	≤ 10	2 - 10	6.4

In the present study amongst pulmonary tubercular disease samples, 39/42 had ADA levels above 40 U/L, while 27/30 of non-tuberculous pleural fluid had ADA levels no more than 40 U/L. In extra-pulmonary disease, tubercular samples 33/36 of ascitic fluid, 18/21 of synovial fluid and 48/54 of CSF had ADA levels above the cutoff, while in non-tubercular samples 72/72 of ascitic fluid, 6/9 of synovial fluid and 63/66 of CSF had values below the cutoff (Table 5).

**Table 5:** Sensitivity, Specificity, Negative and Positive Predictive Values of ADA Estimation in Different Groups

Type of Disease	Aspirated Fluid	Etiology According to Set Criteria	Cut Off Value of ADA	Number of True Positive/Negative Cases	Sensitivity %	Specificity %	Positive Predictive Value %	Negative Predictive Value %
Pulmonary (n = 72)	Pleural (n = 72)	Tuberculous (n = 42)	> 40	39	92.80	90.00	92.86	90.00
		Non-tuberculous (n = 30)	≤ 40	27				
Extra-Pulmonary (n = 258)	Over all includes;	Tuberculous (n = 111)	> 40 / 10	99	94.29	92.16	89.00	95.92
		Non-tuberculous (n = 147)	≤ 40 / 10	141				
	Ascitic (n = 108)	Tuberculous (n = 36)	> 40	33	100	96	91.67	100.00
		Non-tuberculous (n = 72)	≤ 40	72				
	Synovial (n = 30)	Tuberculous (n = 21)	> 40	18	85.71	66.67	85.71	66.67
		Non-tuberculous (n = 09)	≤ 40	06				
	CSF (n = 120)	Tuberculous (n = 54)	> 10	48	84.12	91.30	88.89	95.46
		Non-tuberculous (n = 66)	≤ 10	63				

In extra-pulmonary disease, tubercular samples 99/111 had ADA levels above the cutoff, while in non-tubercular samples 141/147 had below the cutoff level (Table 5). Sensitivity, specificity, positive predictive values and negative predictive values were calculated using standard formulae.

In our study, in cases with pleuropulmonary disease sensitivity was found to be 92.80%, specificity was 90.00%, positive predictive value 92.86% and negative predictive value 90.00%. In cases of extra-pulmonary disease with the involvement of peritoneum, sensitivity was found to be 100.00%, specificity was 96.00%, positive predictive value 91.67% and negative predictive value 100.00%; in cases of musculoskeletal disease, sensitivity was found to be 85.71%, specificity was 66.67%, positive predictive value 85.71% and negative predictive value 66.67%; while in cases of meningeal involvement, sensitivity was found to be 84.12%, specificity was 91.30%, positive predictive value 88.89% and negative predictive value 95.46% (Table 5).

In extra-pulmonary disease, the overall sensitivity was found to be 94.29%, specificity was 92.16%, and positive predictive value 89.00% and negative predictive value 95.92% (Table 5).

## Discussion

Demonstration of AFB, culture, CT and MRI scan etc. are the various means to confirm the etiology of tuberculosis but visualization of AFB in direct smears or in cultures is usually difficult [9] and in most cases negative. Newer methods such as those involving the amplification of bacterial DNA by the PCR and comparable systems, are not available for widespread use in the developing countries. Routine laboratory findings may not be helpful to differentiate tuberculous etiology from other causes.

Cell-mediated response is the predominant form of immune response to tuberculous infection, while both cell-mediated and humoral immune responses are elicited by most non-tuberculous infections in human body. Thus ADA activity, a marker of T-cell activation and cell-mediated immune response can help differentiate tubercular etiology from non-tubercular.

Piras et al were first to report high ADA in tubercular pleural effusion [10]. Subsequently several workers explored its efficacy in the diagnosis of tuberculosis [11] and determined that pleural fluid ADA level less than 40 U/L virtually excludes the diagnosis of tuberculosis [3]. Meta-analysis of studies conducted between 1966 and 1999 concluded that the test performance was reasonably good [12] (sensitivity range 47.1 - 100%, and specificity 0 - 100%) in diagnosing tuberculosis etiology in pleural effusion.

Ocana et al in 1983 [11] and Valdes et al in 1993 [13] and 1996 [14, 15] reported 100% sensitivity and specificity, positive predictive value and negative predictive value in larger sample size studies.

Perez [16] reported the sensitivity and specificity for ADA to be 77 - 100% (average, 93.3%) and 81 - 97% (Average, 91.3%) respectively on summarizing eleven studies, and Chen et al [17] reported the sensitivity and specificity for ADA as 79 - 100% (average, 88.6%) and 80.5 - 96% (average, 85.4%) respectively on summarizing the results of eight studies.

In 2007, a systematic review of ADA by the NGS Health Technology Assessment Programme concluded that there is no evidence to support the use of ADA tests for the diagnosis of pulmonary TB. However, there is considerable evidence to support their use in pleural fluid samples for diagnosis of pleural TB, where sensitivity was very high, and to a slightly lesser extent for TB meningitis. In both pleural TB and TB meningitis, ADA tests had higher sensitivity than any other tests [18].

CSF-ADA estimation was reported to be useful in diagnosing TBM and can differentiate TBM from other neurological disorders [19]. CSF-ADA estimation is a useful method to differentiate TBM from aseptic meningitis [5]. Other researchers have also observed the usefulness of ADA activity in the diagnosis of tubercular disease [20, 21].

Other causes of increase in ADA activity include bacterial infections, rheumatic disease and lymphoproliferative disorders. This article reviews ADA estimation as an effective diagnostic criterion for tuberculous and non-tuberculous disease in pleural, ascitic, synovial fluids and CSF.

In our study, in extra-pulmonary disease with the involvement of peritoneum, sensitivity was found to be 100.00%, specificity 96.00%, positive predictive value 91.67% and negative predictive value 100.00%; in cases of involvement of musculo-skeletal disease, sensitivity was found to be 85.71%, specificity 66.67%, positive predictive value 85.71% and negative predictive value 66.67%; while in cases of meningeal involvement, sensitivity was found to be 84.12%, specificity 91.30%, positive predictive value 88.89 % and negative predictive value 95.46%.

In extra-pulmonary disease, our overall sensitivity was found to be 94.29%, specificity 92.16%, positive predictive value 89.00% and negative predictive value 95.92%; and in pulmonary disease, sensitivity was found to be 92.80%, specificity 90.00%, positive predictive value 92.86 % and negative predictive value 90.00%.

We have observed a statistically significant difference in ADA levels of tubercular and non-tuberculous etiology (p < 0.01). Results of our study indicated that ADA levels in the aspirated fluid are of considerable value in differentiating between tubercular and non-tubercular disease with fairly high accuracy and sensitivity at the taken cutoff.

In TB endemic countries like India, patients with raised ADA levels could be a signal for tuberculosis where AFB positivity is low. This may help in early diagnosis and institution of ATT, thus can significantly alter not only the spread but also the progress and prognosis of TB in our country. Our study is a unique study debating the significance of ADA estimation in diverse form of extra-pulmonary TB including musculoskeletal tuberculosis.

ADA estimation can also be of considerable help in western countries where other causes of lymphocyte rich effusions should be considered like coccidiodomycosis and histoplasmosis. ADA levels, in these cases, are found to be below the cutoff value and thus save the patient from unnecessary anti-tubercular treatment.

It can be concluded that ADA estimation is not only effective but also simple, inexpensive, quick to perform test; it can be used as an alternative to biopsy/culture and also as a guide for further sophisticated investigations. This test may find a place as a routine investigation in the coming days for early detection of tuberculosis where other means are expensive.

In conclusion, ADA estimation is not only a fairly sensitive and specific test (more than 90%), helpful in differentiating tubercular from non-tubercular etiology both in pulmonary and extra-pulmonary disease, but is also simple, inexpensive and rapid. For this reason this test may help in early diagnosis, improve the prognosis and reduce spread of disease and sequlae.
